# Baltic Sea sediments record anthropogenic loads of Cd, Pb, and Zn

**DOI:** 10.1007/s11356-020-10735-x

**Published:** 2020-09-29

**Authors:** Sina Shahabi-Ghahfarokhi, Sarah Josefsson, Anna Apler, Karsten Kalbitz, Mats Åström, Marcelo Ketzer

**Affiliations:** 1 Department of Biology and Environmental Science, Faculty of Health and Life Sciences, Linnaeus University, Stuvaregatan 2, 39231 Kalmar, Sweden; 2 Geological Survey of Sweden, Uppsala, Sweden; 3 Soil Resources and Land Use, Institute of Soil Science and Site Ecology, Dresden University of Technology, Dresden, Germany

**Keywords:** Marine pollution, Heavy metals, Baltic Sea, Geochemistry, Cadmium, Zinc, Lead

## Abstract

The unsustainable settlement and high industrialization around the catchment of the Baltic Sea has left records of anthropogenic heavy metal contamination in Baltic Sea sediments. Here, we show that sediments record post-industrial and anthropogenic loads of Cd, Zn, and Pb over a large spatial scale in the Baltic Sea. We also demonstrate that there is a control on the accumulation of these metals in relation to oxic/anoxic conditions of bottom waters. The total concentrations of Cd, Zn, and Pb were obtained with the near-total digestion method in thirteen cores collected from the Bothnian Bay, the Bothnian Sea, and the west and central Baltic Proper. The lowest average concentrations of Cd, Zn, and Pb were observed in Bothnian Bay (0.4, 125, 40.2 mg kg^−1^ DW, respectively). In contrast, the highest concentrations were observed in the west Baltic Proper (5.5, 435, and 56.6 mg kg^−1^ DW, respectively). The results indicate an increasing trend for Cd, Zn, and Pb from the early nineteenth century until the 1970s, followed by a decrease until 2000–2008. However, surface sediments still have concentrations above the pre-industrial values suggested by the Swedish EPA (Cd is 0.2, Zn is 85, and Pb is 31 mg kg^−1^ DW). The results also show that the pre-industrial Cd, Zn, and Pb concentrations obtained from 3 cores with ages < 1500 B.C. were 1.8, 1.7, and 1.2 times higher, respectively, than the pre-industrial values suggested by the Swedish EPA. To conclude, accumulations of metals in the Baltic Sea are governed by anthropogenic load and the redox conditions of the environment. The significance of correct environmental governance (measures) can be illustrated with the reduction in the pollution of Pb, Zn, and Cd within the Baltic Sea since the 1980s*.*

## Introduction

Anthropogenic metal inputs are reported from seas and coastlines worldwide, e.g., in the East China Sea (Daoji and Daler 2004), Caspian Sea (Bastami et al. 2015; Abadi et al. 2019), Yumurtalik coast (Aytekin et al. 2019), North-East Atlantic (Rodrigues et al. 2009), Greenland Sea (Neff 2002a), North Sea, Mediterranean, Black Sea, and Baltic Sea (Tornero and Hanke 2016). Heavy metals can be toxic to humans/animals and are incorporated into the sea food web through water, food, and sediments (Neff 2002a; Tchounwou et al. 2012; Aytekin et al. 2019). Therefore, heavy metals such as cadmium (Cd), lead (Pb), and zinc (Zn) are frequently studied and monitored to avoid negative impacts on society (Skerfving et al. 1999; Tchounwou et al. 2012) and ecosystems (Neff 2002a; b; c; Aytekin et al. 2019). In Northern Europe, the unsustainable settlement in the catchment of the Baltic Sea region has turned this waterbody into one of the most polluted seas and dead zones in the world (Borg and Jonsson 1996; Conley et al. 2002; Carstensen et al. 2014a). With noticeable pollution in the past 50 years, the 1974 European Regional Sea Conventions (Tornero and Hanke 2016), specifically the HELCOM convention, have taken several actions. These actions have been taken to protect and reduce the pollution of the Baltic Sea (HELCOM 2018), including pollution by heavy metals.

Two common heavy metals associated with health risks in the Baltic Sea region are Pb and Cd. Both metals are reported to cause severe damage to organs (kidney), and Pb also causes neurobehavioral and neurophysiological disease (Skerfving et al. 1999). In contrast, Zn is an essential element for humans and animals (Remeikaitė-Nikienė et al. 2018). However, under long-term high exposure conditions, Zn might cause iron/copper deficiency and neuron-related illness (Ciubotariu et al. 2015). In addition, when Zn is compared to Cd and Pb, it shows a higher affinity to accumulate in marine organisms and tissues (Aytekin et al. 2019). According to the Swedish Environmental Protection Agency (EPA), the pre-industrial values for coastal and sea sediments are based on pre-industrial observations, with values of 0.2, 85, and 31 mg kg^−1^ dry weight (DW) for Cd, Zn, and Pb, respectively (Swedish EPA 2000) (Appendix Table 7). Cadmium, Zn, and Pb concentrations above these values have been reported in several studies focusing on separate parts of the Baltic Sea, Bothnian Bay (Leivuori and Niemistö 1995; Perttilä 2003; Vallius 2014), Gulf of Finland (Vallius 2014), and Gdansk and Baltic Proper (Suess and Erlenkeuser 1975; Perttilä 2003; Zaborska 2014). In addition, previous studies reported maximum concentrations of Cd, Zn, and Pb in the 1970s (Leivuori and Niemistö 1995; Borg and Jonsson 1996; Zaborska 2014).

Studies have reported the effects of climate change, the long-term tendency (early twentieth century) of increasing anoxic areas of bottom waters, and the excessive anthropogenic nutrient/organic loads in the deep and central parts of the Baltic Sea (Borg and Jonsson 1996; Conley et al. 2002; Kabel et al. 2012; Carstensen et al. 2014a; b; Mohrholz 2018). These imposed changes in redox conditions influence the biogeochemical transformations of the suspended particulate matter (SPM) (Beldowski et al. 2010), colloids (Neff 2002b), and soluble metal forms within the water column and sediments (Borg and Jonsson 1996; Neff 2002a; b; c). In addition, metal concentrations in sediments can correlate with grain size surface charge density, cation exchange capacity (CEC), and specific surface area (SSA) (Neff 2002b; Fukue et al. 2006). Anoxic conditions release adsorbed metals from the surface of iron (Fe)/manganese (Mn) hydroxides, and they instead form metal sulfides (Neff 2002c; Beldowski et al. 2010). However, contrary to the assumptions of Borg and Jonsson (1996) and Rogan Šmuc et al. (2018) report higher concentrations of Pb from reduced (anoxic) sediments related to the formation of stable Fe and Mn complexes. Cadmium and Zn are also under the influence of oxic/anoxic conditions (Pohl and Hennings 1999; Neff 2002a; b). Under oxic conditions, Cd is released when organic matter (OM) is mineralized (Neff 2002a; Rogan Šmuc et al. 2018), while Zn is adsorbed to Fe/Mn oxides and OM (Neff 2002b). Overall, Cd, Zn, and Pb are reported to be more abundant in oxic than anoxic waters (Öztürk 1995; Pohl and Hennings 1999); additionally, the foremost soluble Cd, Zn, and Pb species in anoxic waters originate from the bisulfide complexes of these metals (Öztürk 1995; Neff 2002c).

In this report, the overall aim was to study Cd, Zn, and Pb metal pollution in the sediments of the Baltic Sea, focusing on anomalies in the different basins of the Baltic Sea using the same field and analytical methods. This approach will enable us to have a nonbiased comparison among different basins of the Baltic Sea. A specific aim was to identify anthropogenic loads and understand the processes that control the quantity and distribution of Cd, Pb, and Zn in sediments of the Gulf of Bothnia (Bothnian Bay and Bothnian Sea), Åland Sea, and Baltic Proper. Furthermore, the effects of oxic/anoxic conditions in the bottom waters of the Baltic Sea and their relation to observed metal concentrations in sediments are studied. In addition, this study will compare the obtained results of pre-industrial Cd, Zn, and Pb from the aforementioned basins to the pre-industrial values published by the Swedish EPA (Swedish EPA 2000). The study of metal concentration trends will further assist us in evaluating how effective measures to reduce concentrations are over time. Overall, we expect to contribute to the understanding of Cd, Zn, and Pb in sediments under oxic/anoxic conditions, which is important with regard to future climate change- and eutrophication-related anoxic growth in the Baltic Sea and similar water bodies.

## Methods

**Fig. 1:**
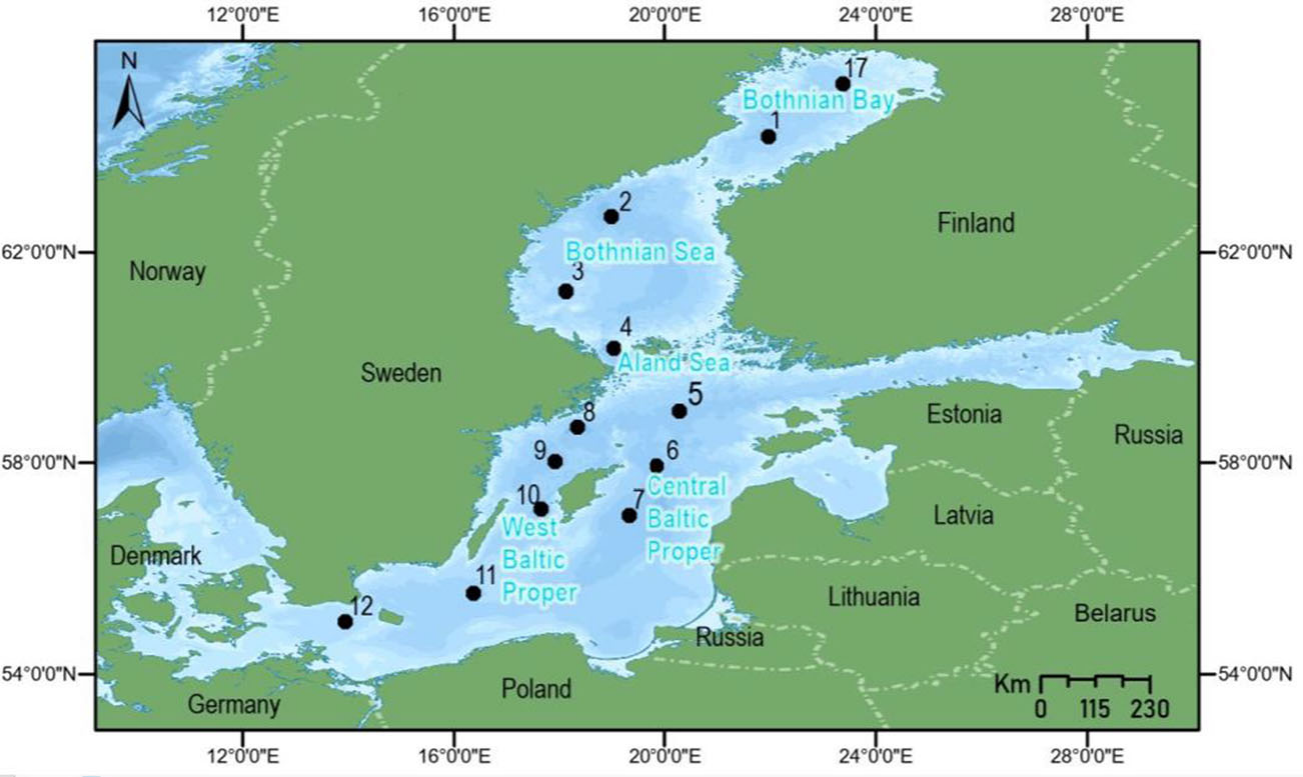
Map of the Baltic Sea showing the location of the 13 cores (numbered 1–12 and 17) used in this study. Image processed and adapted under GNU Free Documentation License. Image is georeferenced by ArcGIS. The graticule lines, north direction, station locations, and name of countries and seas are added to Original Image. In addition, color of lands and borderlines are modified

A total of 137 sediment samples were obtained during the Swedish Environmental Monitoring Programme of offshore sediments in 2008, which was operated by the Geological Survey of Sweden on behalf of the Swedish Environmental Protection Agency (Apler and Josefsson 2016). The collected cores were from 13 stations, including two in Bothnian Bay (stations 17 and 1), three in the Bothnian Sea and Åland Sea (stations 2–4), three in the central Baltic Proper (stations 5–7), and five in the west Baltic Proper (stations 8–12) (Apler and Josefsson 2016) (Fig. 1 and Table 1). Sediment cores were taken with a Gemini gravity corer (Niemistö 1974) onboard the S/V Ocean Surveyor vessel between May and August 2008 (Apler and Josefsson 2016). Cores were sliced into 1-cm intervals (Appendix Tables 2, 3, 4, and 5), freeze-dried, and ground by mortar and pestle. Sediment accumulation rates and the age of each slice were estimated (Appendix Tables 2, 3, 4, and 5) from Cesium-137 determination in sediments (Josefsson and Apler 2019). However, not all slices could be included in this study due to insufficient sample weights for total metal analysis (Table 1 and Appendix Tables 2, 3, 4, and 5).

**Table 1:** Geographical coordinates, water depth, and the number of slices per core (N) obtained from each of the 13 cored monitoring stations in the Baltic Sea used in this study

Area	Core	Latitude	Longitude	N	Depth (m)
Bothnian Bay	17	N 65° 11′ 26.69″	E 23° 23′ 48.01″	10	87
	1	N 64° 11′ 47.84″	E 21° 59′ 28.88″	8	113
Bothnian Sea and Åland Sea	2	N 62° 41′ 0.29″	E 18° 59′ 39.24″	9	200
	3	N 61° 16′ 5.64″	E 18° 8′ 16.12″	11	79
	4	N 60° 11′ 30.26″	E 19° 2′ 48.40″	11	230
Central Baltic Proper	5	N 58° 58′ 38.16″	E 20° 18′ 25.42″	9	175
	6	N 57° 56′ 30.99″	E 19° 52′ 9.63″	10	195
	7*	N 57° 0′ 47.91″	E 19° 20′ 4.35″	13	173
West Baltic Proper	8	N 58° 40′ 41.25″	E 18° 21′ 24.45″	10	403
	9*	N 58° 1′ 29.74″	E 17° 55′ 45.23″	12	178
	10*	N 57° 8′ 0.08″	E 17° 40′ 6.04″	14	111
	11	N 55° 32′ 35.91″	E 16° 23′ 23.56″	11	70
	12	N 54° 59′ 55.29″	E 13° 57′ 29.60″	8	47

*Cores that were used to determine background values in this report

All 137 samples were analyzed for the total concentrations of Cd, Zn, and Pb. Sample preparation included digestion of a 250-mg dry weight (DW) sample with stepwise addition of hydrofluoric, nitric, and perchloric acids. The obtained solution was dried until incipient dryness was achieved, and subsequently, the solution phase was retained by the addition of aqua regia. Metal concentrations were determined by inductively coupled plasma mass spectrometry at Activation Laboratories Ltd. (ActLabs), Canada (http://www.actlabs.com/, Appendix Table 6). The precision of the analytical method was controlled with the coefficient of variation (Appendix Table 6) using anonymously randomized duplicates (Gill 2014).

**Fig. 2:**
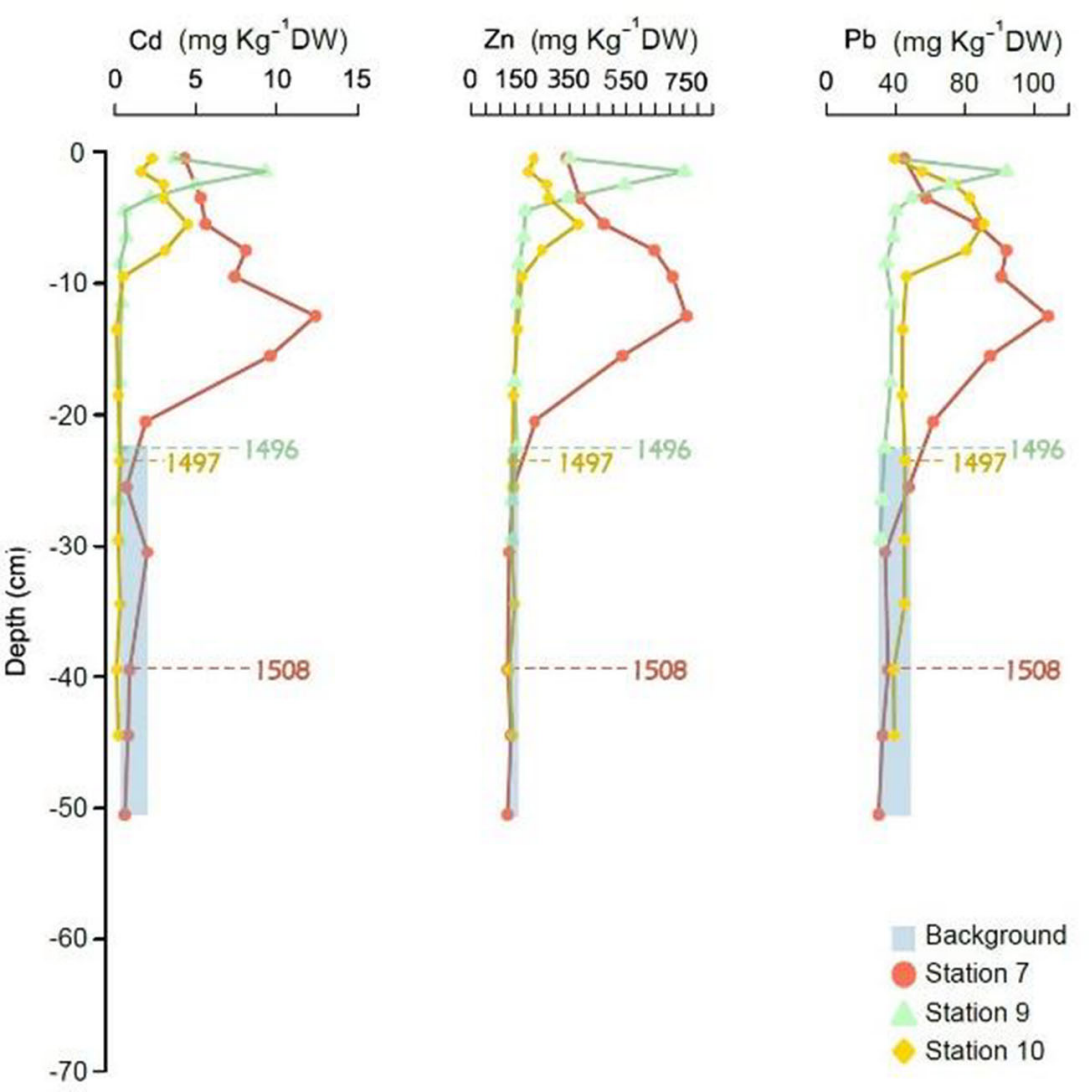
Concentration (mg kg^−^1 DW) profiles for Cd, Zn, and Pb in the three cores with the lowest sediment accumulation rates which were selected for determination of pre-industrial concentrations. Numbers on the figures are the calculated depositional years of the sediment slice at that depth, based on dating using Caesium-137. See Fig. 1 and Table 1 for core location

The calculation of pre-industrial levels of metals in sediments was based on the quality and quantity of data (Gałuszka and Migaszewski 2011; Zgłobicki et al. 2011; de Paula Filho et al. 2015). The direct method, also known as the geochemical method, is most frequently used (Gałuszka and Migaszewski 2011; Zgłobicki et al. 2011; de Paula Filho et al. 2015) and was thus used in this study. This method is based on visualization and relies on samples that date back to (i.e., are older than) the industrial revolution and are thus largely free of human influence (Zgłobicki et al. 2011). Visually, the quasi-linear, invariable concentration trend with depth in old profiles was recognized (Gałuszka and Migaszewski 2011; Zgłobicki et al. 2011). Pre-industrial metal median concentration (Gałuszka and Migaszewski 2011) values were considered in the three cores (stations 7, 9, and 10 located in the Baltic Proper) with the lowest sedimentation rates (< 0.08 cm/year), which contained sediments > 500 years of age (Fig. 2, Appendix Tables 4 and 5). ArcGIS was used to georeference and plot sampling points in the Baltic Sea image. In addition, the images were developed/edited in Affinity Designer Software version 1.7.3.481. The graphs and statistical analysis were created and conducted in R Studio (2018).

## Results

### Pre-industrial Cd, Zn, and Pb concentrations

The median and standard deviation (SD) of Cd, Zn, and Pb concentrations from sediments > 500 years old are 0.25 (0.21), 141.5 (9.05), and 36.3 (6.23) mg kg^−1^ DW, respectively (Fig. 2, and Appendix Tables 4, 5, and 7). These values are based on cores located in the Baltic Proper, areas where low sediment accumulation rates allow the sampler to reach sufficiently old samples in the sediment. These calculated pre-industrial concentrations are 1.25, 1.7, and 1.2 times, respectively, higher than the Cd, Zn, and Pb pre-industrial values suggested by the Swedish Environmental Protection Agency (Swedish EPA 2000) (Appendix Table 7).

### Anthropogenic loads of Cd, Zn, and Pb

#### Bothnian Bay

The average surface sample (0–1 cm) concentrations in cores 17 and 1, collected in northern and southern Bothnian Bay, exceed the calculated pre-industrial values by 0.9, 52.5, and 32.7 mg kg^−1^ DW for Cd, Zn, and Pb, respectively. At station 17, the concentration peaks correspond to the early ca. 1980s and are as high as 1.1, 176, and 78.2 mg kg^−1^ DW for Cd, Zn, and Pb, respectively. The sediment accumulation rates of stations 17 and 1 are similar; however, at station 1, Cd and Zn continue to increase and are highest in the surface sample, corresponding to ca. year 2000, with values of 1.3 and 217 mg kg^−1^ DW, respectively. In contrast, the concentrations of Pb at station 1 decrease from 124 mg kg^−1^ DW in the samples from ca. 1980s to 91 mg kg^−1^ DW in the topmost sample corresponding to ca. 2000.

#### The Bothnian Sea and Åland Sea

The average surface sample (0–1 cm) concentrations in cores 2, 3, and 4 collected in the northern and southern Bothnian Sea and the Åland Sea exceed the calculated pre-industry values by 0.1, 31.5, and 2 mg kg^−1^ DW for Cd, Zn, and Pb, respectively. Below this depth, in the top 10 cm and towards the surface, the concentrations decreased, although it should be noted that at station 2, only one sample was analyzed from these depths, which makes the trend uncertain (Fig. 4). All three stations peak at depths of 10–20 cm, corresponding roughly to the early 1980s (Fig. 4 and Appendix Table 3). The Cd concentrations decreased strongly below a peak at 26–27 cm at station 2 (1.1 mg kg^−1^ DW). The corresponding pattern for Zn was similar between stations 2 and 4, while concentrations were lower at station 3; however, for Pb, station 4 generally had the highest concentration (Fig. 4).

#### Central Baltic Proper

The average surface concentrations (0–1 cm) in cores 5–7 collected in the central Baltic Proper exceed the calculated pre-industrial values by 3.6 and 710 mg kg^−1^ DW for Cd and Zn, respectively. However, the average Pb concentrations from the surface samples (Fig. 5) were lower than the calculated pre-industrial value of 4.9 mg kg^−1^ DW. In stations of the central Baltic Proper, the sediment accumulation rates were highly variable and decreased in the order of stations 5 > 6 > 7 (Fig. 5 and Appendix Table 4). All three cores showed a similar trend of strongly decreasing metal concentrations towards the surface. At stations 6 and 7, this decrease is observed from the nineteenth century onward, and the decrease was observed at station 5 from an undefined age. At station 5, as the concentrations increased to the bottom of the core, the maximum values may not have been reached due to high sediment accumulation rates. The metal concentrations reached high values in all cores, up to approximately 10–15 mg kg^−1^ DW Cd, 700–800 mg kg^−1^ DW Zn, and 100–120 mg kg^−1^ DW Pb (Fig. 5 and Appendix Table 5). The highest Cd and Zn concentrations occurred at station 5, with values as high as 14.7 and 819 mg kg^−1^ DW, respectively, in approximately the 1950s. The highest Pb concentration occurred at station 7, with a value as high as 128 mg kg^−1^ DW, before 1895.

#### West Baltic Proper

The average surface concentrations (0–1 cm) in cores 8–12 collected in the west Baltic Proper exceed the calculated pre-industrial values by 2.4, 112, and 114.5 mg kg^−1^ DW for Cd, Zn, and Pb, respectively. Stations 8–12 show a decreasing concentration trend in the surface samples, although the values still exceed the calculated pre-industrial values at most stations (Fig. 6a, b and Appendix Table 7). For stations 9, 11, and 12, the highest concentrations are present in the sediment layer sampled below the surface layer, and no long-term decreasing trend is thus apparent. For stations 8 and 10, the peak concentrations are further down in the core. The metal concentrations reached high values in all cores, up to approximately 10 mg kg^−1^ DW Cd, 700–800 mg kg^−1^ DW Zn, and 100–120 mg kg^-1^ DW Pb (Fig. 6a, b and Appendix Table 5). The highest Cd concentrations occurred at station 8, with a value as high as 10.4 mg kg^−1^ DW in approximately the early 1970s. The highest Zn and Pb concentrations occurred at stations 9 and 12, respectively, with maximum values of 753 mg kg^−1^ DW and 114 mg kg^−1^ DW, respectively, in approximately the mid-1970s.

## Discussion

### Pre-industrial values

**Fig. 3:**
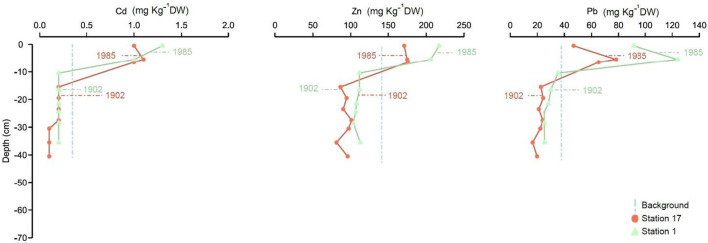
Concentration (mg kg^−1^ DW) profiles of Cd, Zn, and Pb from stations 17 and 1 in the North and South of the Bothnian Bay. Indicated ages are calculated from sediment accumulation rates based on Caesium-137 concentrations in sediments. Median calculated background values obtained for cores 7, 9, and 10 are plotted for reference (dashed vertical lines). See Fig. 1 and Table 1 for core location

Differences between the calculated pre-industrial concentrations for Cd, Pb, and Zn in this study and the Swedish EPA (Swedish EPA 2000) show the complex geochemistry of marine sediments (Reimann and Garrett 2005; Gałuszka and Migaszewski 2011). It is important to note that the pre-industrial values established by the Swedish EPA are median values for the entire marine environment of Sweden and thus do not take any regional differences in, e.g., bedrock chemistry or redox conditions, into account. Fukue et al. (2006) report anomalies in pre-industrial metal concentrations related to sorption/adsorption capacity and the characteristics of the specific sediment sample. Additionally, pre-industrial Zn and Pb concentrations show different values when compared to gneiss bedrock (Fukue et al. 2006). The highest concentrations of Zn are also visible in the longer cores, which might be an indicator of the dominant feature of the bedrock (Fukue et al. 2006) and the possible presence of Zn within heavy minerals (Neff 2002b). Fukue et al. (2006) also consider grain size, CEC and SSA as factors affecting the observed concentration anomalies from pre-industrial values in sediment cores. In this study, samples with Cd, Zn, and Pb concentrations lower (Fig. 3) than the calculated pre-industrial values for the Baltic Proper cores with an age < 1500 B.C. (Fig. 2) might be explained by the difference in the texture, bedrock, CEC, SSA (Fukue et al. 2006), and oxic/anoxic conditions (Carstensen et al. 2014a).

### Declining trends towards the surface

In this study, peak concentrations are mainly found around the 1970s and 1980s (with the exception of stations 6 and 7, see Fig. 5). Borg and Jonsson (1996) also reported elevated concentrations of Cd, Zn, and Pb from Baltic Sea sediments dating back to 1970 and later. However, most of the studied locations show a recovery trend after the 1980s, which aligns with the HELCOM reports, suggesting the efficiency of policies limiting emissions of Cd, Zn, and Pb to the environment. This result means that the load and concentrations of pollutants previously introduced in the Baltic Sea from industry and agriculture have been reduced. In the case of Pb, the general decreasing trend starting in the 1980s is attributed to restrictions of Pb-based additives in fuels (Östlund and Sternbeck 2001; Beldowski et al. 2010; Vallius 2014).

**Fig. 4:**
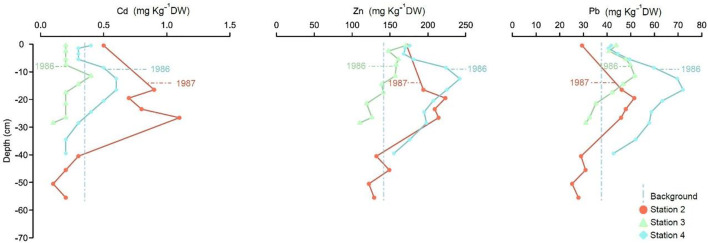
Concentration (mg kg^−1^ DW) profiles of Cd, Zn, and Pb from stations 2–4 belonging to the North and South of the Bothnian Bay and Åland Sea are presented. Indicated ages are calculated from sediment accumulation rates based on Caesium-137 concentrations in sediments. Median calculated background values obtained for cores 7, 9, and 10 are plotted for reference (dashed vertical lines). See Fig. 1 and Table 1 for core location

**Fig. 5:**
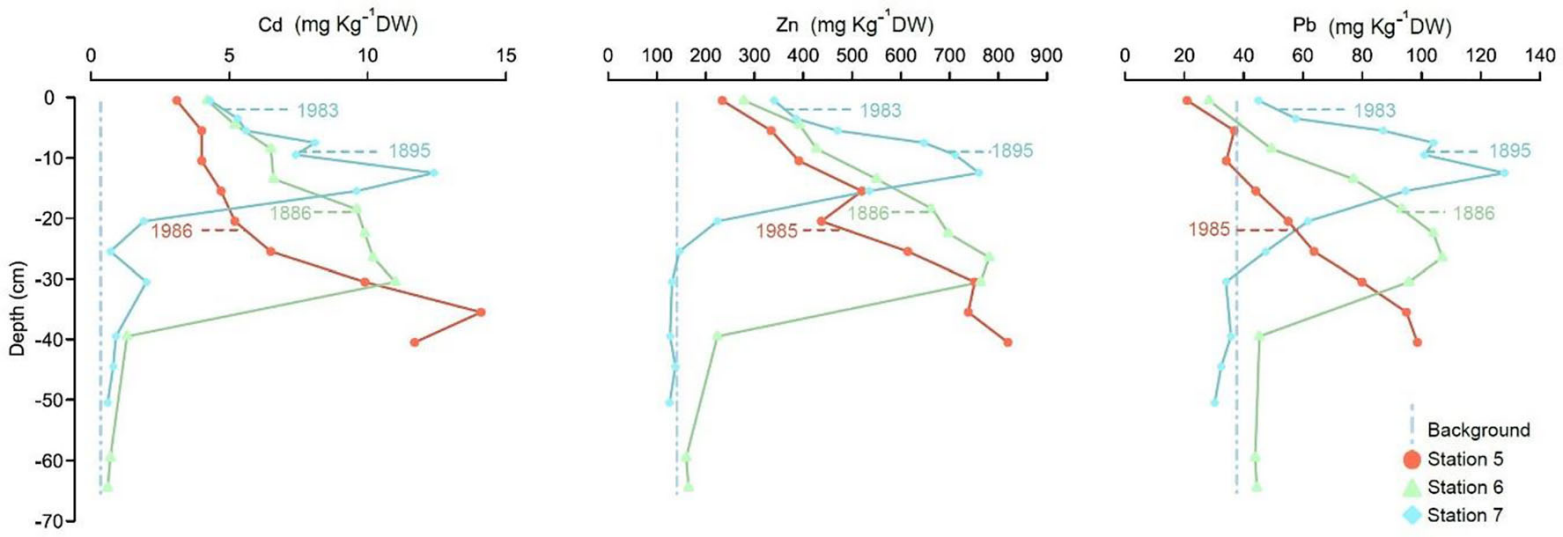
Concentration (mg kg^−1^ DW) profiles of Cd, Zn, and Pb from stations 5–7 belonging to the central Baltic Proper are presented as Central Baltic Proper. Indicated ages are calculated from sediment accumulation rates based on Caesium-137 concentrations in sediments. Median calculated background values obtained for cores 7, 9, and 10 are plotted for reference (dashed vertical lines). See Fig. 1 and Table 1 for core location

**Fig. 6:**
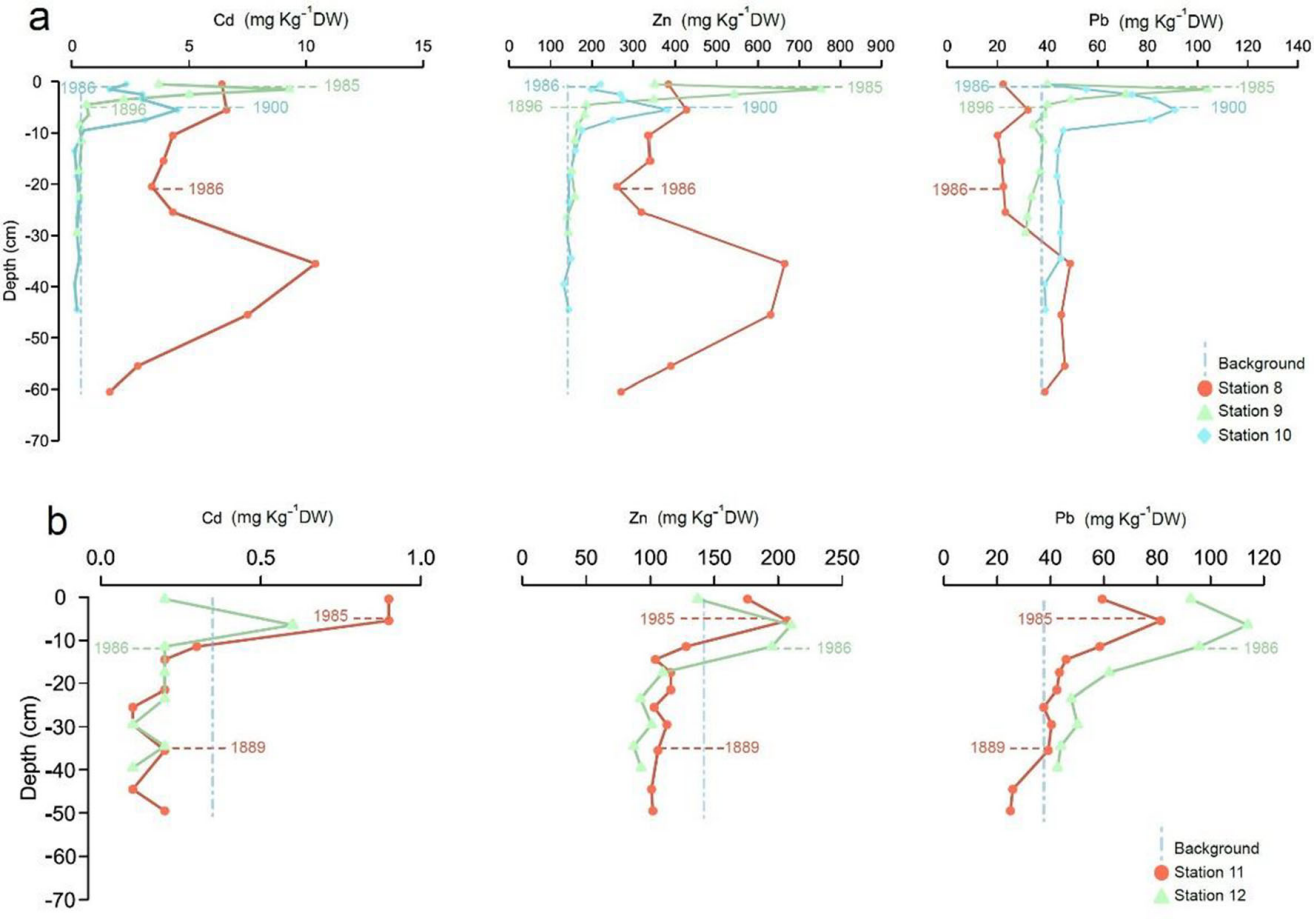
Concentration (mg kg^−1^ DW) profiles of Cd, Zn, and Pb from stations 8–10 (**a**) and 11–12 (**b**) belonging to the central Baltic and west Baltic Proper. Indicated ages are calculated from sediment accumulation rates based on Caesium-137 concentrations in sediments. Median calculated background values obtained for cores 7, 9, and 10 are plotted for reference (dashed vertical lines). See Fig. 1 and Table 1 for core location

All cores from the Gulf of Bothnia and Åland Sea (Figs. 3 and 4) show lower maximum concentrations of Cd, Zn, and Pb than cores from the Baltic Proper (Figs. 5 and 6). Comparing Cd, Zn, and Pb among different basins, the west and central Baltic Proper show higher peaks and concentrations (Fig. 4). These findings agree with previous observations of higher Cd, Zn, and Pb concentrations in the Baltic Proper relative to those in the entire Baltic Sea (Suess and Erlenkeuser 1975; Perttilä 2003; Zaborska 2014). The strong reducing conditions near the sediment-water interface (Carstensen et al. 2014a) might explain the fixation of metal species in anoxic sediments (Borg and Jonsson 1996).

**Fig. 7:**
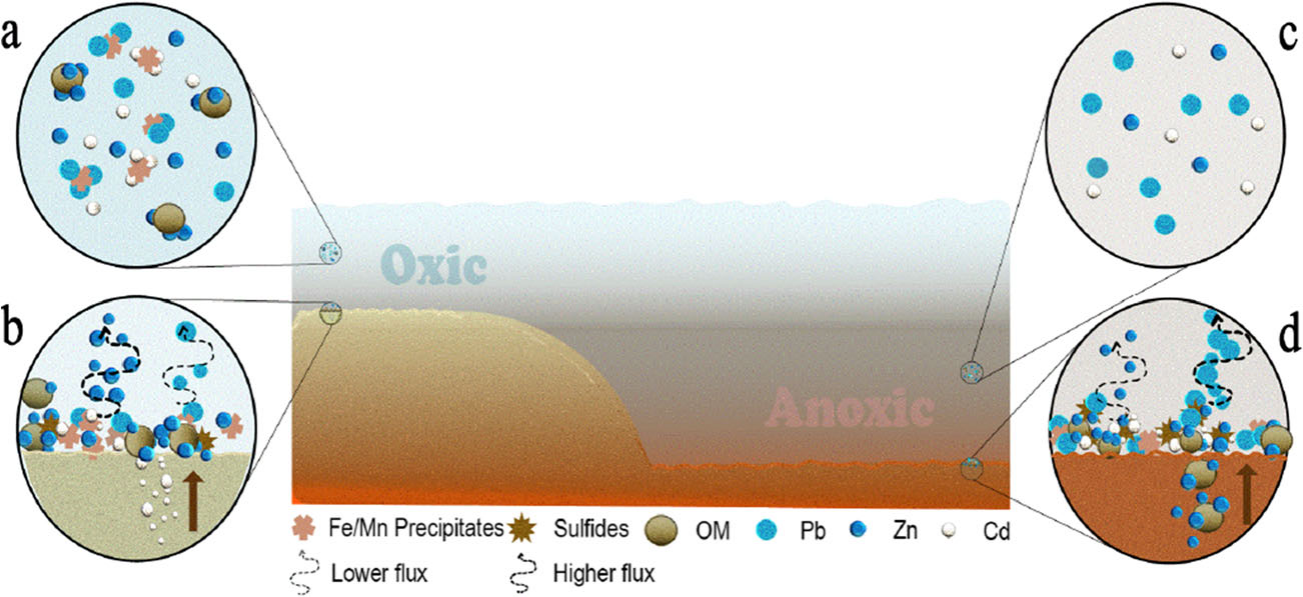
Illustrating the expected behavior of Cd, Zn, and Pb in the Baltic Sea water, pore water, sediments (Mn precipitates, sulfides), and OM under oxic (**a**, **b**) and anoxic conditions (**c**, **d**). Different metals behave in certain ways from dissolution in oxic and anoxic waters (**a**, **c**) and formation of hydroxides in oxic water (**a**, **b**) to precipitation and interacting with sulfides in anoxic water (**d**). Higher concentrations of Cd, Zn, and Pb are present in oxic waters. When oxygen and OM are available, OM mineralization release Cd into solution (**a**) and at bottom sediments the soluble Cd in the pore water interacts with Fe/Mn precipitates (**b**). However, under the same conditions, Zn is bound to OM (**a**) and Pb is attracted towards Mn precipitates in solution (**a**). At the bottom sediments, OM is degraded over time and Zn is released and associated with Mn/Fe oxyhydroxides (**b**). Also, the flux between sediments and the water column tends to be higher for Zn and lower for Pb (**b**). In the anoxic water, in general lower concentrations of Cd, Zn, and Pb are present in solution (**c**) owing its solubility to bisulfides. In the anoxic bottom sediments, Cd and Zn have high affinity towards sulfides in clay-rich sediments and Pb reacts with Mn/Fe forming complexes. The flux from sediment to the water column is faster for Zn and slower for Pb in anoxic waters (**d**). Image is developed in Affinity Designer Software version 1.7.3.481. References (Öztürk 1995; Borg and Jonsson 1996; Pohl and Hennings 1999; Neff 2002a; b; c; Ingri et al. 2014; Rogan Šmuc et al. 2018; Beldowski et al. 2010).

### Gulf of Bothnia

Concerning the general behaviors of Cd, Zn, and Pb in the Gulf of Bothnia (Figs. 3 and 4), the results from this study and previous reports (Leivuori and Niemistö 1995; Vallius 2014; Zaborska 2014) show similar patterns between profiles. Cadmium is reported to be as high as 0.2–2 mg kg^−1^ DW, Zn ranged from 60 to 220 mg kg^−1^ DW, and Pb ranged from 5 to 200 mg kg^−1^ DW (Leivuori and Niemistö 1995; Borg and Jonsson 1996; Perttilä 2003; Vallius 2014), which are in the same range as Pb in this study (Cd 0.1–1.3 mg kg^−1^ DW, Zn 81–242 mg kg^−1^ DW, and Pb 16–124 mg kg^−1^ DW). On average, the water depth is deeper in the Bothnian Sea (65 m) than in Bothnian Bay (40 m) (Håkansson et al. 1996). In addition, the freshwater load and water circulation are higher in Bothnian Bay than in the Bothnian Sea (Håkansson et al. 1996; Perttilä 2003), which oxygenates the bottom waters and brings metals into solution. Overall, Cd, Zn, and Pb are reported to be more abundant in oxic waters than in anoxic waters (Fig. 7a) (Öztürk 1995; Pohl and Hennings 1999). In the case of the Bothnian Sea and Åland Sea, the Cd and Zn concentrations are higher at stations 2 and 4 than at station 1. Stations 2 and 4 show similar concentration anomalies and peak concentrations of Cd and Zn. This result might be an effect of anoxic deep water at stations 2 and 4, which are located at water depths of 200 and 230 m, respectively. However, with shallower and better freshwater circulation in the Gulf of Bothnia, this concentration does not exceed the peak concentrations in the Baltic Proper cores. In addition, the dynamics of the Gulf of Bothnia might allow coarser particles with low CEC and SSA (Radzevièius 2002) to enter the basin and therefore restrict the adsorption of soluble metals (Jönsson et al. 2005). With available metals and previously found ferrohydrate nodules in the southern Bothnian Sea (Borg and Jonsson 1996), which act as adsorption surfaces for soluble metals, higher concentrations of sediment metals are expected in the Bothnian Sea. Increases in Cd concentrations in surface samples under oxic conditions might be explained by OM mineralization (Rogan Šmuc et al. 2018), which releases soluble Cd from the pore water (Rogan Šmuc et al. 2018) that can be transferred to the surface (Rogan Šmuc et al. 2018). With soluble Cd and the presence of Mn precipitates (Pohl and Hennings 1999) and ferrohydrate nodules (Borg and Jonsson 1996), Cd enrichment might occur in the surface layers. Zinc in the water column has a higher affinity to form stable colloidal OM; this colloidal OM has stronger bonds than those of Zn adsorbed to SPM (Neff 2002b). In oxic sediments, OM and Fe/Mn oxides are associated with higher Zn concentrations in bottom sediments (Neff 2002b; Ingri et al. 2014). Moreover, high Zn concentrations are found in phytoplankton, which affects the Zn concentrations of bottom sediments (Ingri et al. 2014) through the degradation of OM followed by Fe/Mn oxide or ferrohydrate nodule uptakes (Fig. 7a). The lower concentrations of Zn in the top layers of oxic sediments (Figs. 3 and 4) could be explained by the faster flux of dissolved Zn from sediments into the oxic water column (Neff 2002b) (Fig. 7b). For lead, close contamination sources and the inland transport of contamination into the Bothnian Bay basin may have caused the higher concentrations reported by Perttilä (2003) than those found in this study. Lead concentrations in the SPM of oxic/anoxic regions are reported to be in the same range as the concentrations of Zn (Öztürk 1995). Additionally, Pb in oxic sediments is reported to be associated with oxic Fe/Mn coatings loaded on clay particles (Neff 2002c). Moreover, the surface concentrations of Cd, Zn, and Pb at station 1 are higher than those in the other cores of the Gulf of Bothnia. Higher surface sample concentrations at this station might be due to high organic matter inputs and direct metal loads from the smelter plant located at Rönnskär, which is close to station 1 (HELCOM 2013), which has been an important pollution source. However, there are industrial source contamination hotspots reported close to all five cores (17 and 1–4) (HELCOM 2013). Stations 17, 1, and 4 might have experienced pollution from the main marine navigation routes (HELCOM 2018) as well as from all main industrial pollution sources (HELCOM 2013). The elevated concentrations of Cd, Pb, and Zn in the Åland Sea core (Fig. 3) can be related to agricultural runoff hotspot pollution (HELCOM 2013) and the high-density traffic line connecting the Gulf of Bothnia and the Baltic Proper (HELCOM 2018).

### Baltic Proper

Concerning the general behavior of Cd, Zn, and Pb in the Baltic Proper, the comparison of metals in the different sectors of the Baltic Proper (Figs. 5 and 6) shows similar patterns in this and previous studies (Leivuori and Niemistö 1995; Zaborska 2014). Cadmium is reported to be as high as 0.1–10.93 kg^−1^ DW, Zn as high as 44–1021 kg^−1^ DW, and Pb as high as 18–> 200 mg kg^−1^ DW (Suess and Erlenkeuser 1975; Perttilä 2003; Zaborska 2014), which are similar to the values in this report (Cd 0.1–14.1 mg kg^−1^ DW, Zn 87–816 mg kg^−1^ DW, and Pb 20–128 mg kg^−1^ DW). In general, the high concentrations of metals in the Baltic Proper might be explained by the high organic matter content in sediments, the presence of sulfides in anoxic environments (Borg and Jonsson 1996), and the relatively longer residence time of anoxic waters (Leivuori and Niemistö 1995). Kabel et al. (2012) also suggest that the anoxic waters of the Baltic Proper can become more widespread with current and future climate change. As mentioned, Cd, Zn, and Pb are influenced by anoxic environments (Fig. 7c). The foremost soluble Cd, Zn, and Pb species in anoxic waters originate from the bisulfide complexes of these metals (Fig. 7c) (Öztürk 1995; Neff 2002c). The higher concentrations of Cd in the Baltic Proper might be related to the available fine particles and clay-rich sediments in deep anoxic waters (Pohl and Hennings 1999). Pohl and Hennings (1999) reported the high affinity of Cd sulfide species to these particles (Fig. 7d). In reduced sediments, Zn has a higher affinity towards OM and sulfides than towards Fe/Mn oxides (Neff 2002b; Ingri et al. 2014). This feature could also be observed in pore waters of anoxic sediments where soluble Zn adsorbs to OM (Neff 2002b) (Fig. 7d). Other mutual geochemical behaviors of Cd and Zn in the Baltic are the complex formations with bi/polysulfides and in less soluble forms in anoxic environments (Fig. 7d) (Pohl and Hennings 1999). These could explain the higher concentrations of Cd and Zn in the sediment profiles of stations 5–10 compared to those of the Gulf of Bothnia and stations 11 and 12. In the Gotland Basin (stations 5–7), studies have revealed the existence of iron sulfides, which can contain high concentrations of trace elements (Perttilä 2003). Stations 8–12, compared to the central Baltic Proper concentrations, have an increase in surface sediment concentrations and peaks that may be related to water depth, such that in deeper areas (station 8), more anoxic conditions exist and favor the formation of metal-organic complexes and sulfides and, as a result, increase the surface sediment concentrations (Borg and Jonsson 1996; Beldowski et al. 2010). Station 8 is close to municipality emission hotspots (HELCOM 2013), which might contribute to the elevated levels of Cd, Zn, and Pb. Station 9 is also very deep, which explains the anoxic conditions favoring the precipitation of metals under anoxic conditions. Stations 11 and 12 show lower surface sediment concentrations as well as lower values than those in other cores in the west Baltic Proper. This result might be explained by the frequent freshwater import from the Bornholm into the Baltic Proper (Mohrholz 2018). The local inputs of fresh waters will increase the oxygen levels of the water in the area and eliminate the reducing conditions that contribute to the precipitation of Mn (Perttilä 2003). This process will favor adsorptions of trace metals onto the surface Mn oxides. For lead, the limited transport of Pb contacting SPM and other sediments into the Baltic Proper basin might explain the restrained concentrations reported by Perttilä (2003) and this study. Lead in anoxic sediments is adsorbed to sulfides, forming a predominant and stable form (Neff 2002c). However, with excessive amounts of sulfides, Pb forms unstable complexes (Neff 2002c). In addition, Borg and Jonsson (1996) and Rogan Šmuc et al. (2018) report higher concentrations of Pb from reduced (anoxic) sediments, which is related to the formation of stable Fe and Mn complexes (Fig. 7d). However, lead is also reported to be highly unstable in anoxic sediments, with higher flux from the sediments to the water column (Fig. 7d) (Neff 2002c). This result could explain the higher concentrations of Pb in the topmost sediments of the Gulf of Bothnia and in the shallow sites (stations 11 and 12) of the Baltic Proper (Figs. 3, 4, 5, and 6).

## Conclusion

This report illustrates the peak concentrations of Cd, Zn, and Pb that date back to the 1970–1980s. The reported concentrations of metals in surface sediments (0–1 cm) of the Gulf of Bothnia and Baltic Proper are higher than the calculated pre-industrial values. The calculated pre-industrial values in this study for the Baltic Proper are elevated compared to the overall pre-industrial values established by the Swedish EPA. A trend of improvement, possibly related to actions taken by the countries around the Baltic Sea, is present. Stations deviated from this trend, increasing close in the surface layer, which might be explained by surface water interactions with the sediments. Varying metal concentrations in the Baltic Sea are partly related to redox conditions promoted by climate change, limitation of oxic water mixing, and eutrophication. In addition, the presence of point contamination sources, Mn nodules, sulfides, and Mn/Fe oxides contribute to these concentrations. The higher concentrations of Cd, Zn, and Pb observed in the Baltic Proper and some part of the Gulf of Bothnia are also related to the reducing conditions of the deep waters in this area, altering metal forming complexes and resulting in precipitation. However, the precipitation and dilution of metals such as Pb will need further investigation with regard to oxic/anoxic waters. Thus, it could be concluded that anoxic sea waters, which tend to become more important with the advance of eutrophication and climate change, may contribute to higher metal concentrations in sediments.

## Appendix

**Table 2:** The depth and the estimated age of each slice that is analyzed for Cd, Zn, and Pb in stations 17 and 1 (belonging to the Bothnian Bay)

Station	Depth (cm)	Year	Cd (mg kg^−1^ DW)	Zn (mg kg^−1^ DW)	Pb (mg kg^-1^ DW)
17	0–1	2002	1	171	46.8
	5–6	1975	1.1	175	78.2
	10–11	1947	1	176	65.3
	15–16	1919	0.2	86.3	22.6
	18–19	1902	0.2	94.5	24.3
	23–24	1875	0.2	89.9	21.1
	27–28	1852	0.2	101	24
	32–33	1825	0.1	97.1	22.2
	35–36	1808	0.1	81.1	16.5
	40–41	1780	0.1	95.8	19.8
1	0–1	2002	1.3	217	91.2
	5–6	1971	1	206	124
	10–11	1939	0.2	112	35.3
	16–17	1902	0.2	112	30
	20–21	1877	0.2	108	28.2
	24–25	1852	0.2	107	25.1
	28–29	1827	0.2	104	25.1
	35–36	1783	0.2	113	25.3

**Table 3:** The depth and the estimated age of each slice that is analyzed for Cd, Zn, and Pb in stations 2–4 (belonging to the Bothnian Sea)

Station	Depth (cm)	Year	Cd (mg kg^−1^ DW)	Zn (mg kg^−1^ DW)	Pb (mg kg^−1^ DW)
2	0–1	2006	0.5	172	29.4
	16–17	1983	0.9	194	46.2
	19–20	1978	0.7	223	51.5
	23–24	1972	0.8	209	47.9
	26–27	1968	1.1	214	45.9
	40–41	1947	0.3	132	29
	45–46	1939	0.2	149	30.9
	50–51	1932	0.1	122	25.2
	55–56	1924	0.2	129	27.9
3	0–1	2005	0.2	171	43.9
	2–3	2000	0.2	148	40.8
	5–6	1992	0.2	161	47.9
	7–8	1986	0.2	158	49.8
	11–12	1976	0.4	157	51.7
	14–15	1967	0.3	139	46.7
	17–18	1959	0.2	141	42.1
	21–22	1949	0.2	119	35.2
	26–27	1935	0.2	126	32.6
	28–29	1930	0.1	110	31
4	0–1	2006	0.4	176	41.7
	1–2	2003	0.3	170	40.6
	3–4	1998	0.3	168	44.6
	5–6	1994	0.3	181	49.5
	8–9	1987	0.5	224	59.8
	12–13	1977	0.6	242	69.7
	16–17	1968	0.6	225	72.1
	20–21	1958	0.5	207	63.3
	24–25	1948	0.4	195	58.7
	28–29	1939	0.3	198	57.8
	34–35	1925	0.2	176	52.1
	39–40	1913	0.2	155	42.7

**Table 4:** The depth and the estimated age of each slice that is analyzed for Cd, Zn, and Pb in stations 5–7 (Central Baltic Proper)

Station	Depth (cm)	Year	Cd (mg kg^−1^ DW)	Zn (mg kg^−1^ DW)	Pb (mg kg^−1^ DW)
5	0–1	2007	3.1	234	20.9
	5–6	2002	4	334	36.8
	10–11	1997	4	391	34.1
	16–17	1991	4.7	520	44.1
	21–22	1987	5.2	437	55
	26–27	1982	6.5	614	63.7
	31–32	1977	9.9	751	79.9
	36–37	1972	14.1	738	94.9
	41–42	1967	11.7	819	98.6
6	0–1	2007	4.2	278	28.3
	4–5	2002	5.2	392	38.8
	8–9	1998	6.5	427	49.3
	13–14	1992	6.6	550	77
	18–19	1986	9.6	665	93.3
	22–23	1982	9.9	697	104
	26–27	1977	10.2	781	107
	31–32	1972	11	763	95.7
	39–40	1963	1.3	224	45.3
	59–60	1940	0.7	160	43.9
	64–65	1934	0.6	165	44.4
7	0–1	1996	4.3	340	45
	3–4	1958	5.3	385	57.6
	5–6	1933	5.6	470	87.1
	7–8	1908	8.1	647	104
	9–10	1883	7.4	711	101
	12–13	1846	12.4	760	128
	15–16	1808	9.6	535	94.6
	20–21	1746	1.9	224	61.7
	25–26	1683	0.7	146	47.5
	31–32	1608	2	131	34
	39–40	1508	0.9	127	35.7
	44–45	1446	0.8	138	32.4
	51–52	1358	0.6	126	30.2

**Table 5:** The depth and the estimated age of each slice that is analyzed for Cd, Zn, and Pb in stations 8–12 (West Baltic Proper)

Station	Depth (cm)	Year	Cd (mg kg^−1^ DW)	Zn (mg kg^−1^ DW)	Pb (mg kg^−1^ DW)
8	0–1	2007	6.4	383	22.3
	5–6	2002	6.6	427	32.2
	10–11	1997	4.3	336	20.1
	15–16	1992	3.9	340	21.7
	20–21	1987	3.4	261	22.4
	26–27	1982	4.3	320	23.2
	36–37	1972	10.4	665	49.1
	46–47	1962	7.5	631	45.5
	56–57	1953	2.8	389	46.9
	62–63	1947	1.6	271	38.9
9	0–1	1986	3.7	350	40
	1–2	1964	9.3	753	104
	2–3	1941	5	543	71.3
	3–4	1919	2.2	348	49.5
	4–5	1897	0.6	187	40
	6–7	1852	0.7	183	38.5
	8–9	1808	0.3	166	34.4
	11–12	1741	0.4	159	38.2
	17–18	1608	0.3	151	37.2
	22–23	1497	0.3	159	33.7
	26–27	1408	0.2	140	32
	29–30	1341	0.2	143	31.3
10	0–1	1987	2.3	221	39.4
	1–2	1965	1.6	199	55.5
	2–3	1944	3	270	73.7
	3–4	1923	3	276	82.9
	5–6	1902	4.5	380	90.9
	7–8	1838	3.1	251	81
	9–10	1795	0.5	176	46.3
	13–14	1710	0.1	160	44.1
	18–19	1604	0.2	146	43.8
	23–24	1497	0.3	144	45.5
	29–30	1370	0.2	139	45.2
	34–35	1263	0.3	150	45.2
	39–40	1157	0.1	132	38.9
	44–45	1051	0.2	144	39.2
11	0–1	2005	0.9	176	59.4
	5–6	1991	0.9	207	81.4
	11–12	1975	0.3	128	58.5
	14–15	1966	0.2	104	45.9
	17–18	1958	0.2	116	43.4
	21–22	1947	0.2	116	42.4
	25–26	1936	0.1	103	37.5
	29–30	1925	0.1	113	40.4
	35–36	1908	0.2	106	39.2
	44–45	1883	0.1	101	25.9
	49–50	1869	0.2	102	25
12	0–1	2006	0.2	137	92.6
	6–7	1996	0.6	210	114
	11–12	1987	0.2	195	95.8
	17–18	1976	0.2	110	62.1
	23–24	1966	0.2	92.4	47.8
	29–30	1955	0.1	101	50.2
	34–35	1947	0.2	87.1	43.9
	39–40	1938	0.1	92.8	42.7

**Table 6:** The quality control measurements for Cd, Zn, and Pb using the ICP-MS method. The estimates of precision given by the coefficient of variation (CV%)

Quality control measurements of the analyzed elements			
Analyte symbol	Cd	Zn	Pb
Unit	ppm	ppm	ppm
Detection limit	0.1	0.2	0.5
Upper limit	1000	10,000	5000
Method	TD-MS	TD-MS	TD-MS
CV%	4.8	2.3	2.9
GXR-4 Meas	< 0.1	63.1	47.3
GXR-4 Cert	0.86	73	52
GXR-4 Meas	< 0.1	68.9	51
GXR-4 Cert	0.86	73	52
GXR-6 Meas	< 0.1	126	101
GXR-6 Cert	1	118	101
GXR-6 Meas	< 0.1	136	106
GXR-6 Cert	1	118	101
OREAS 97 (4 Acid) Meas	–	579	132
OREAS 97 (4 Acid) Cert	–	646	147
OREAS 97 (4 Acid) Meas	–	590	138
OREAS 97 (4 Acid) Cert	–	646	147
OREAS 97 (4 Acid) Meas	–	637	153
OREAS 97 (4 Acid) Cert	–	646	147
OREAS 98 (4 Acid) Meas	–	1310	288
OREAS 98 (4 Acid) Cert	–	1360	345
DNC-1a Meas	–	64.5	6
DNC-1a Cert	–	70	6.3
SBC-1 Meas	0.4	202	37.2
SBC-1 Cert	0.4	186	35
OREAS 45d (4-Acid) Meas	–	41.3	37.2
OREAS 45d (4-Acid) Cert	–	45.7	35
OREAS 45d (4-Acid) Meas	–	37.3	21.8
OREAS 45d (4-Acid) Cert	–	45.7	21.8
OREAS 96 (4 Acid) Meas	–	387	21
OREAS 96 (4 Acid) Cert	–	457	21.8
OREAS 96 (4 Acid) Meas	–	434	91.2
OREAS 96 (4 Acid) Cert	–	457	101
Method Blank	< 0.1	< 0.2	< 0.5

**Table 7:** Values (min, max, average, median, and SD) for the background values calculated for this study as well as values from the Swedish EPA

Background values						
Metal	This study (mg kg^−1^ DW)					EPA** (mg kg^−1^ DW)
	Min	Max	Average	Median	SD*	
Cd	0.1	2	0.35	0.25	0.21	0.2
Zn	126	159	141.5	141.5	9.05	85
Pb	30	47	37.7	36.3	6.23	31

*Standard deviation (SD) of the mean

**Reference value—Swedish Environmental Protection Agency (Swedish EPA 2000)
